# Diminazene as a Potential Pharmacological Candidate Against Tebuconazole-Induced Cardiotoxicity

**DOI:** 10.3390/biomedicines14102245

**Published:** 2026-10-04

**Authors:** Ana Luiza Borges Resende, Fabiana dos Santos Martins, Ivan Lobo de Sousa Marques, Alessandra Silva Teles, Douglas Lamounier de Almeida, Antônio Rogério Dias Guimarães, Artur Santos-Miranda, Daniella Santos Nascimento, Diego Santos de Souza, Lucas Miranda Kangussu, Julliane Vasconcelos Joviano-Santos

**Affiliations:** 1 Faculdade de Ciências Médicas de Minas Gerais, Belo Horizonte 30130-110, MG, Brazil; 2 Laboratório de Investigações NeuroCardíacas, Ciências Médicas de Minas Gerais (LINC CMMG), Belo Horizonte 30130-110, MG, Brazil; 3 Department of Physiology and Biophysics, Universidade Federal de Minas Gerais, Belo Horizonte 31270-901, MG, Brazil; 4 Post-Graduate Program in Health Sciences, Faculdade de Ciências Médicas de Minas Gerais, Belo Horizonte 30130-110, MG, Brazil; 5 Laboratório de Inovação Técnico-Científica, FELUMA-Ciências Médicas de Minas Gerais, Belo Horizonte 30130-007, MG, Brazil; 6 Department of Physiology, Universidade Federal de Sergipe, Aracajú 49107-230, SE, Brazil; 7 Department of Morphology, Universidade Federal de Minas Gerais, Belo Horizonte 31270-901, MG, Brazil

**Keywords:** tebuconazole, diminazene, renin-angiotensin system, cardiotoxicity, heart, cardioprotective drugs

## Abstract

**Background/Objectives**: Tebuconazole (TEB) is a fungicide whose exposure has been increasingly linked to adverse cardiovascular outcomes. However, no specific therapeutic approaches are available to counteract TEB-induced cardiotoxicity, underscoring the need for new pharmacological strategies. Diminazene (DIZE) has emerged as a promising cardioprotective agent through its modulatory effects on the renin-angiotensin system (RAS), particularly via activation of the ACE2/Ang-(1–7)/Mas receptor. **Methods**: This study investigated whether DIZE protects against TEB cardiotoxicity using an in vitro model utilizing H9c2 cardiomyoblasts and an ex vivo model employing Langendorff-perfused rat hearts. **Results**: In cardiomyoblasts, TEB induced a dose-dependent reduction in cell viability, accompanied by increased apoptotic/necrotic cell death. A concentration of 100 µM TEB, representing an intermediate toxicity profile, was selected for subsequent experiments. Co-exposure to DIZE (100–1000 µM) attenuated TEB-induced cytotoxicity in a concentration-dependent manner, restored cell viability and reduced oxidative and nitrosative stress markers, specifically reactive oxygen species (measured via DHE) and nitric oxide (via DAF-2 DA). Furthermore, TEB showed lower mean ACE2 expression and DIZE prevented the downregulation. In isolated heart preparations, TEB caused pronounced mechanical and electrophysiological dysfunction. Mechanically, TEB reduced left ventricular developed pressure, impaired contractility, increased end-diastolic pressure, and prolonged systolic duration. Electrophysiologically, TEB induced bradycardia, PR prolongation, and QT/QTc and T-wave prolongation, indicating delayed atrioventricular conduction and ventricular repolarization. DIZE markedly attenuated both the mechanical and electrical abnormalities induced by TEB. **Conclusions**: These findings provide the first experimental evidence of DIZE against TEB-induced cardiac injury and support further investigation of its potential against pesticide-induced cardiotoxicity.

## 1. Introduction

Tebuconazole (TEB) is a triazole fungicide extensively used in agriculture owing to its high efficacy, systemic activity, and plant growth-regulating properties [1,2]. Due to its broad-spectrum antifungal activity, TEB is widely applied in cereals, vegetables, fruits, medicinal plants, and ornamental species, making it one of the most commercially important fungicides worldwide [2,3]. Its activity is mediated through inhibition of sterol biosynthesis via blockade of lanosterol-14α-demethylase, leading to disruption of fungal membrane integrity and impaired cellular growth [4]. However, the same physicochemical stability and widespread applicability that underlie its agricultural success also raise concerns regarding chronic environmental persistence and human exposure, particularly in populations with occupational or environmental risk. 

Human exposure to TEB is widespread and occurs through multiple routes, including inhalation during agricultural application, dermal absorption, and ingestion of contaminated food and water [2,5]. TEB can accumulate systemically, a characteristic linked to multi-organ toxicity and metabolic disturbances [5]. Supporting the biological plausibility of these adverse effects, a physiologically based toxicokinetic model validated across multiple species, including humans, has demonstrated that TEB is readily absorbed and distributed under realistic exposure scenarios, providing an important framework for estimating internal human doses and health risks [6].

Among the systemic effects of TEB, accumulating evidence indicates significant risks to cardiovascular health. At the cellular level, TEB disrupts cardiac energy metabolism by inhibiting mitochondrial biogenesis and impairing oxidative phosphorylation [7]. It also activates programmed cell death pathways through p53 upregulation and apoptosis mediated by caspase-9 and caspase-3 [8]. In cardiomyoblasts, TEB induces dose-dependent DNA fragmentation and cell cycle dysregulation, reinforcing its cytotoxic potential [8]. Beyond structural damage, TEB exerts marked electrophysiological toxicity. Acute exposure has been shown to alter the electromechanical properties of murine heart and human induced pluripotent stem cell-derived cardiomyocytes through multichannel blockade [9], modulate sodium currents mediated by human NaV1.5 channels [10,11], and impair cardiac electrical function in vivo, thereby increasing susceptibility to arrhythmias [12]. Collectively, these findings identify TEB as a multifaceted cardiotoxic agent capable of compromising both myocardial integrity and cardiac electrical stability.

Despite these growing concerns, no specific therapeutic strategies or clinical guidelines exist for the management of TEB-induced cardiotoxicity. This gap highlights the need to identify novel pharmacological agents capable of mitigating pesticide-induced cardiac injury. In this context, the repurposing of non-conventional drugs has emerged as a promising strategy [13,14]. Diminazene Aceturate (DIZE), an aromatic diamidine traditionally used in the treatment of trypanosomiasis [15,16], is a particularly strong candidate due to its independent cardioprotective properties. DIZE has been widely reported to enhance angiotensin-converting enzyme 2 (ACE2) activity and ACE2-associated cardioprotective signaling, an important counterregulatory pathway within the renin–angiotensin system (RAS) [17], involved in cardiovascular homeostasis, fluid balance, and cellular function [18,19,20]. Beyond RAS modulation, DIZE can exhibit direct myocardial effects, improving cardiac function, reducing infarct size, attenuating ventricular remodeling, enhancing cardiac electrical activity, and promoting cardioprotection in ischemia/reperfusion injury models [21,22,23,24,25,26]. Importantly, DIZE has also demonstrated acute antiarrhythmic properties in vitro, attributed to direct modulation of cardiomyocyte contractility and excitability [27], supporting a direct cellular mechanism of action, and suggesting its potential as a redox- and function-modulating therapeutic agent.

Despite these promising properties, the cardioprotective mechanisms of DIZE remain incompletely understood, particularly in the context of pesticide-induced cardiotoxicity. While the cardiotoxic effects of TEB and the cardioprotective actions of DIZE have each been described independently, no studies to date have evaluated whether DIZE can attenuate the cardiac dysfunction induced by TEB exposure. Addressing this gap will not only enhance our understanding of the mechanisms underlying pesticide-related cardiac injury but also provide initial evidence supporting the repurposing of DIZE and the RAS as a targeted therapeutic strategy. Therefore, the present study investigated whether DIZE attenuates TEB-induced cardiotoxicity through an integrated assessment of cell viability, oxidative stress, apoptosis, contractile function, and electrophysiological alterations.

## 2. Materials and Methods

### 2.1. In Vitro Experimental Model

The H9c2 rat cardiomyoblast cell line (ATCC CRL-1446™) was kindly provided by the Cardiovascular Physiology Laboratory, Department of Physiology and Biophysics, Federal University of Minas Gerais. The cell line was originally obtained from the American Type Culture Collection (ATCC, Manassas, VA, USA) and was expanded and maintained under standard culture conditions. H9c2 cardiomyoblasts were cultured in Dulbecco’s Modified Eagle Medium (DMEM) (Invitrogen, Burlington, ON, USA) supplemented with 4.5 g/L d-(+)-glucose, 1 g/L glutamine, 10% (*v*/*v*) fetal bovine serum, and 1% (*v*/*v*) penicillin/streptomycin. Cultures were maintained at 37 °C in a humidified incubator with 5% CO_2_, and subculturing was performed twice a week, with 3–4-day intervals between passages to avoid overconfluence, as described [10]. Cells were exposed for 24 h to the following reagents: Tebuconazole (% > 97, sc-204906B, Santa Cruz Biotechnology, Santa Cruz, CA, USA) (1, 10, 100, and 1000 µM) and Diminazene Aceturate (% > 98, sc-205651, Santa Cruz Biotechnology, Santa Cruz, CA, USA) (1, 10, 100, and 1000 µM). The concentrations of TEB and DIZE utilized in this study were specifically selected to serve as an in vitro mechanistic and toxicological challenge.

### 2.2. Cytotoxicity

Cells were plated at an appropriate density (1 × 10^4^ to 1 × 10^5^ cells/cm^2^ to reach ~80% confluency within 24 to 48 h) and exposed to test compounds for 24 h. At the end of treatment, cells were collected, washed with PBS, and resuspended in Annexin V binding buffer. Approximately 1 × 10^5^ cells were incubated with Annexin V-FITC and propidium iodide (PI, 1–5 µg/mL) for 15 min at room temperature in the dark, then diluted in binding buffer and analyzed within 1 h using a flow cytometer (BD FACSCanto II flow cytometer, BD Biosciences, San Jose, CA, USA) equipped with a 488-nm laser. Compensation was set using single-stained controls, and 10,000 gated singlet events were acquired per sample. Data were expressed as percentages of viable (Annexin V^−^/PI^−^), early apoptotic (Annexin V^+^/PI^−^), late apoptotic (Annexin V^+^/PI^+^), and necrotic (Annexin V^−^/PI^+^) cells [28].

### 2.3. Detection of Nitric Oxide (NO) and Reactive Oxygen Species (ROS)

Specific fluorescent probes were used to detect and quantify NO and ROS in H9c2 cells after 24 h of exposure to TEB and DIZE. Intracellular NO production was measured using 5 µM 4-amino-5-methylamino-2′,7′-difluorofluorescein diacetate (DAF-2 DA; excitation/emission wavelengths of 495/515 nm, FITC filter set), a sensitive probe for endogenously produced NO. Cytosolic ROS levels were assessed using 5 µM dihydroethidium (DHE; excitation/emission wavelengths of 488/610 nm, PE/Rhodamine filter set), which primarily reacts with superoxide anions to produce a fluorescent signal proportional to their concentration. Cells were incubated with each probe for 30 min at 37 °C in the dark. After incubation, cells were washed once with PBS and maintained in Tyrode’s solution. Fluorescence images were acquired and signal quantification was performed using ImageJ software (Version 1.54g, National Institutes of Health, Bethesda, MD, USA). All procedures were conducted in triplicate from different subcultures at room temperature under minimal light exposure. The investigator responsible for image analysis was unaware of the experimental groups during measurements, and group identification was performed only after completion of the analysis.

### 2.4. Western Blotting

Protein expression in H9c2 cells was evaluated by Western blotting. Briefly, protein samples were extracted using an appropriate lysis buffer supplemented with protease inhibitors, and protein concentration was determined. Equal amounts of total protein (15 µg) were mixed with Laemmli sample buffer, denatured, and separated by SDS-PAGE. Following electrophoresis, the resolved proteins were transferred onto PVDF membranes. Membranes were subsequently blocked with 5% albumin diluted in Tris-buffered saline containing 0.1% Tween-20 (TBST) for 1 h at room temperature. After blocking, membranes were incubated overnight at 4 °C with a rabbit polyclonal anti-ACE2 antibody (GTX15349; GeneTex, Zeeland, MI, USA), diluted in 1% albumin in TBST at the concentration recommended by the manufacturer (0.5–2 µg/mL). Following incubation, membranes were washed with TBST and incubated with an appropriate HRP-conjugated anti-rabbit IgG secondary antibody, diluted in 1% albumin in TBST, for 1 h at room temperature. After further washing with TBST, immunoreactive bands were visualized using an enhanced chemiluminescence (ECL) detection system and captured using a digital imaging system. GAPDH (D16H11; Cell Signaling, Danvers, MA, USA) was used as a loading control to assess relative protein loading and normalize ACE2 expression. ACE2 and GAPDH signals were quantified by densitometric analysis using ImageJ software (Version 1.54g, National Institutes of Health, Bethesda, MD, USA).

### 2.5. Isolated Hearts Preparation

Male Wistar rats weighing 250–300 g were used for the ex vivo experiments. All animal procedures were approved by the ethics committee (#3273170524; Animal Use Ethics Committee; Ethics Committee of Federal University of Sergipe). After heparin (1000 IU, i.p., 15 min), the rats were euthanized, and the hearts were rapidly excised and mounted on a constant-flow aortic perfusion apparatus based on the Langendorff technique, with a perfusion flow of 10 mL/min. Hearts were mounted on a Langendorff apparatus and retrogradely perfused at constant pressure with oxygenated Krebs–Henseleit solution (95% O_2_/5% CO_2_). Following a 20-min stabilization period, hearts were assigned to one of four experimental groups: Control, DIZE, TEB, or TEB+DIZE. TEB at 100 µM and DIZE at 100 µM were perfused for 12 min. In the combined treatment group, TEB and DIZE were administered simultaneously throughout the entire perfusion period; DIZE was not used as a rescue intervention. Hearts were randomly assigned to the experimental groups. Exclusion criteria for isolated hearts were established according to the recommendations already described [29], including technical complications during cannulation, prolonged ischemic time, inadequate coronary perfusion, or unstable baseline cardiac function before the experimental protocol. The temperature was maintained at 37 ± 0.1 °C using a Haake F3 thermostat system (Thermo Haake, Karlsruhe, Germany). Left ventricular developed pressure (LVDP) was measured by inserting a water-filled balloon into the left ventricular cavity, connected to a pressure transducer (MLT0699/A). The system was calibrated using a mercury column (150 mmHg), and parameters were analyzed over 2 min of consecutive beats. Pressure signals were amplified with a Bridge Amp FE221 (ADInstruments, Dunedin, New Zealand) and converted via an analog–digital converter (PowerLab 4/35, ADInstruments). For electrocardiogram (ECG) recordings, three Ag/AgCl/NaCl (1 M) electrodes were immersed to detect the heart’s macroscopic electrical activity. The preparation was allowed to stabilize for 20 min before data acquisition. The QT interval was corrected for heart rate using the rodent-normalized Bazett formula (QTc-B = QT/(RR/f)), with the average RR interval of the control group (0.222 ± 0.039 s) used as the normalization factor, as previously described [30,31]. All signals were amplified and digitized at a 4 kHz sampling rate using a PowerLab 4/35 data acquisition system (ADInstruments); recordings and analyses were performed using LabChart version 8.0 Pro software (ADInstruments).

### 2.6. Statistical Analysis

Statistical analyses were performed using GraphPad Prism software (version 8.0; GraphPad Software, San Diego, CA, USA). Data normality was assessed using the Kolmogorov–Smirnov test. For normally distributed data, multi-group comparisons were evaluated using either one-way ANOVA followed by Tukey’s post hoc test or two-way ANOVA followed by Bonferroni’s multiple comparisons test, as appropriate. For non-normally distributed data, the Kruskal–Wallis test was applied followed by Dunn’s post-test. A significance level of 5% (*p* < 0.05) was adopted for all statistical evaluations. Data were expressed as mean ± standard error of the mean (SEM).

## 3. Results

### 3.1. TEB Induces Dose-Dependent Cytotoxicity That Is Attenuated by DIZE

**Figure 1:**
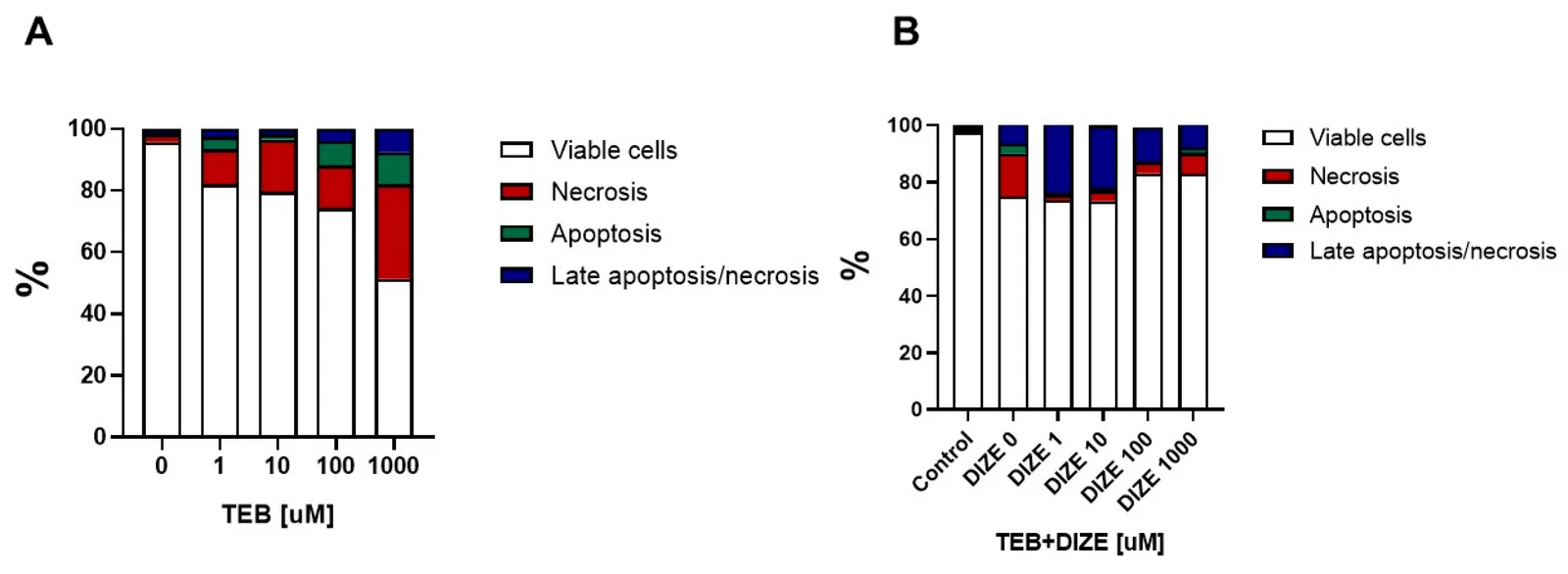
Dose-dependent cytotoxic effects of Tebuconazole (TEB) and modulation by Diminazene (DIZE). (**A**) Cells were exposed to increasing concentrations of TEB, and cell viability, necrosis, apoptosis, and late apoptosis/necrosis were assessed. TEB induced a dose-dependent decrease in cell viability, accompanied by a progressive increase in markers of necrosis and apoptosis. (**B**) Cells were co-exposed to TEB (100 µM) and increasing concentrations of DIZE (1–1000 µM). Low concentrations of DIZE (1–10 µM) resulted in a modest improvement in cell viability, whereas higher concentrations (100–1000 µM) provided more substantial protection against TEB-induced cytotoxicity. Data are expressed as percentage of total cells from triplicates. The independent experiment was considered the biological replicate.

Exposure to TEB induced a dose-dependent reduction in cell viability, which was accompanied by a progressive increase in markers of necrosis, early apoptosis, and late apoptosis (Figure 1A). The most pronounced cytotoxic effects were observed at 1000 µM, whereas 100 µM produced an intermediate response and was therefore selected for subsequent experiments. When cells were co-exposed to TEB and DIZE (Figure 1B), the cell death profile was modified: low concentrations of DIZE (1–10 µM) conferred a modest improvement in viability, while higher concentrations (100–1000 µM) provided more substantial protection against TEB-induced cytotoxicity.

### 3.2. Evaluation of DIZE Effects on Cellular Oxidative Stress

**Figure 2:**
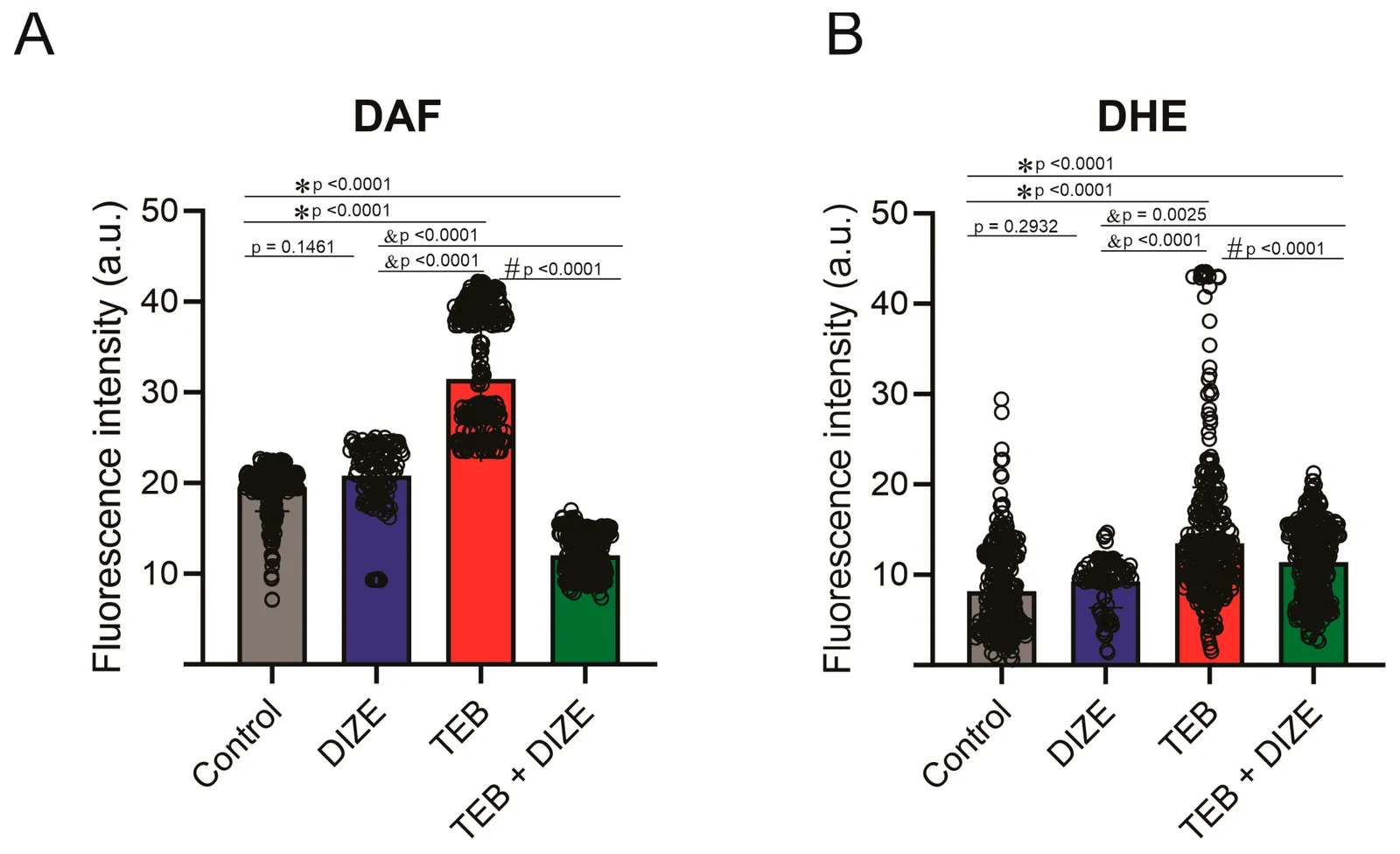
Effects of Tebuconazole (TEB) and Diminazene (DIZE) on nitric oxide (NO) and reactive oxygen species (ROS) production. (**A**) Quantitative fluorescence analysis of NO levels using the DAF probe. (**B**) Quantitative fluorescence analysis of ROS production using the DHE probe. Data were analyzed using one-way ANOVA followed by Tukey’s post hoc test. Data are expressed as mean ± SEM; * *p* < 0.05 vs. control, & *p* < 0.05 vs. DIZE; # *p* < 0.05 vs. TEB. Individual cell measurements are shown as individual points. A total of 300 cells were analyzed across three independent experiments (100 cells per condition per experiment). The independent experiment was considered the biological replicate (*n* = 3 per group), and statistical analyses were performed using the experiment-level data; Kruskal–Wallis test was applied followed by Dunn’s post-test.

Quantitative fluorescence analyses revealed that TEB exposure significantly increased intracellular NO and ROS production, as detected by DAF and DHE probes, respectively. Regarding NO detection by DAF (Figure 2A), treatment with TEB (100 µM) significantly increased fluorescence intensity compared with the control group (control: 19.56 ± 0.34 a.u.; TEB: 31.49 ± 0.53 a.u.; *p* < 0.0001), indicating enhanced NO availability. Treatment with DIZE alone did not significantly alter DAF fluorescence compared with the control group (DIZE: 20.81 ± 0.34 a.u.; *p* = 0.1461). In contrast, co-exposure to TEB and DIZE markedly reduced fluorescence intensity (TEB + DIZE: 12.01 ± 0.13 a.u.), with values significantly lower than those observed in both the TEB group and the control group (*p* < 0.0001).

Similarly, analysis of ROS using the DHE probe (Figure 2B) showed a pronounced increase in fluorescence in the TEB group compared with the control group (control: 8.18 ± 0.28 a.u.; TEB: 13.48 ± 0.36 a.u.; *p* < 0.0001), confirming increased ROS accumulation following TEB exposure. Treatment with DIZE alone did not significantly alter DHE fluorescence compared with the control group (DIZE: 9.25 ± 0.31 a.u.; *p* = 0.2932). However, co-exposure with TEB and DIZE significantly attenuated the TEB-induced increase in ROS fluorescence (TEB + DIZE: 11.40 ± 0.25 a.u.), with values significantly lower than those observed in the TEB group (*p* < 0.0001), although still significantly higher than both the control group (*p* < 0.0001) and the DIZE group (*p* = 0.0025). Together, these findings indicate that DIZE alone does not significantly affect intracellular ROS levels, whereas DIZE co-exposure partially mitigates the TEB-induced accumulation of ROS, supporting a potential protective or modulatory effect of DIZE against TEB-induced oxidative stress.

### 3.3. Effects of TEB and DIZE on ACE2 Protein Expression

**Figure 3:**
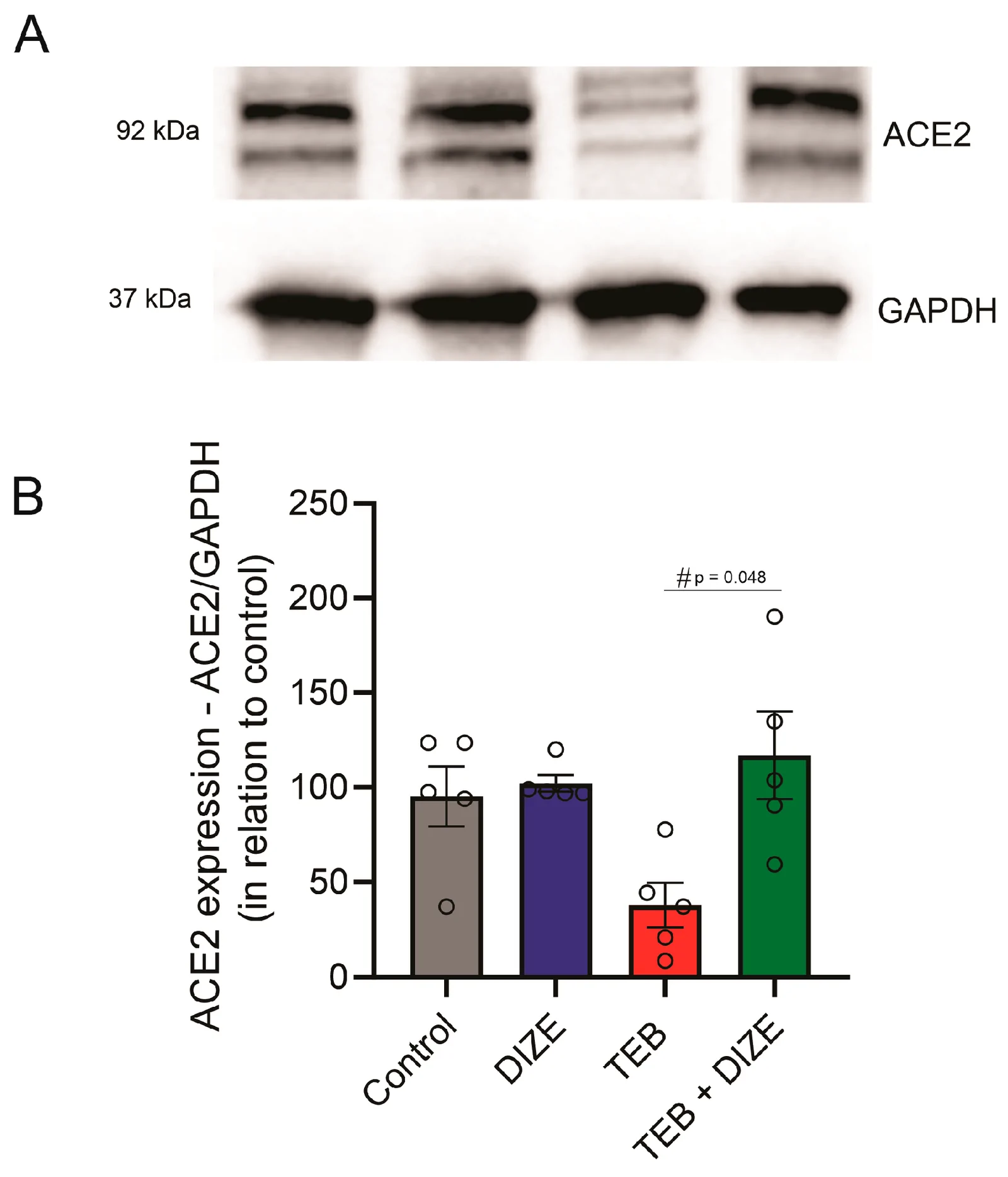
Effect on ACE2 protein expression after Tebuconazole (TEB) and Diminazene (DIZE) treatment. (**A**) Representative Western blot images for ACE2 (92 kDa) and the endogenous control GAPDH (37 kDa). (**B**) Densitometric analysis of ACE2 protein expression. The only significant difference was between TEB and TEB + DIZE groups. Values were normalized to GAPDH expression and are represented relative to the Control group (%), *n* = 5 per group; Data are expressed as mean ± SEM, # *p* < 0.05 vs. TEB; Kruskal–Wallis test was applied followed by Dunn’s post-test.

ACE2 protein expression was assessed via Western blot to determine the effects of the DIZE intervention (Figure 3A). While exposure with TEB alone resulted in the lowest average levels of ACE2 (37.84 ± 11.79%), this reduction did not reach statistical significance when compared to the Control (*p* = 0.1694) or DIZE (*p* = 0.0894) groups. Notably, the only statistically significant difference observed in this experiment was between the TEB and the combination (TEB + DIZE) groups. The combined treatment (TEB + DIZE) significantly increased ACE2 expression (116.90 ± 23.13%) when compared exclusively to the TEB-treated group (*p* = 0.0485) (Figure 3B).

### 3.4. DIZE Attenuated the Deleterious Effects of TEB on Ventricular Contractility

**Figure 4:**
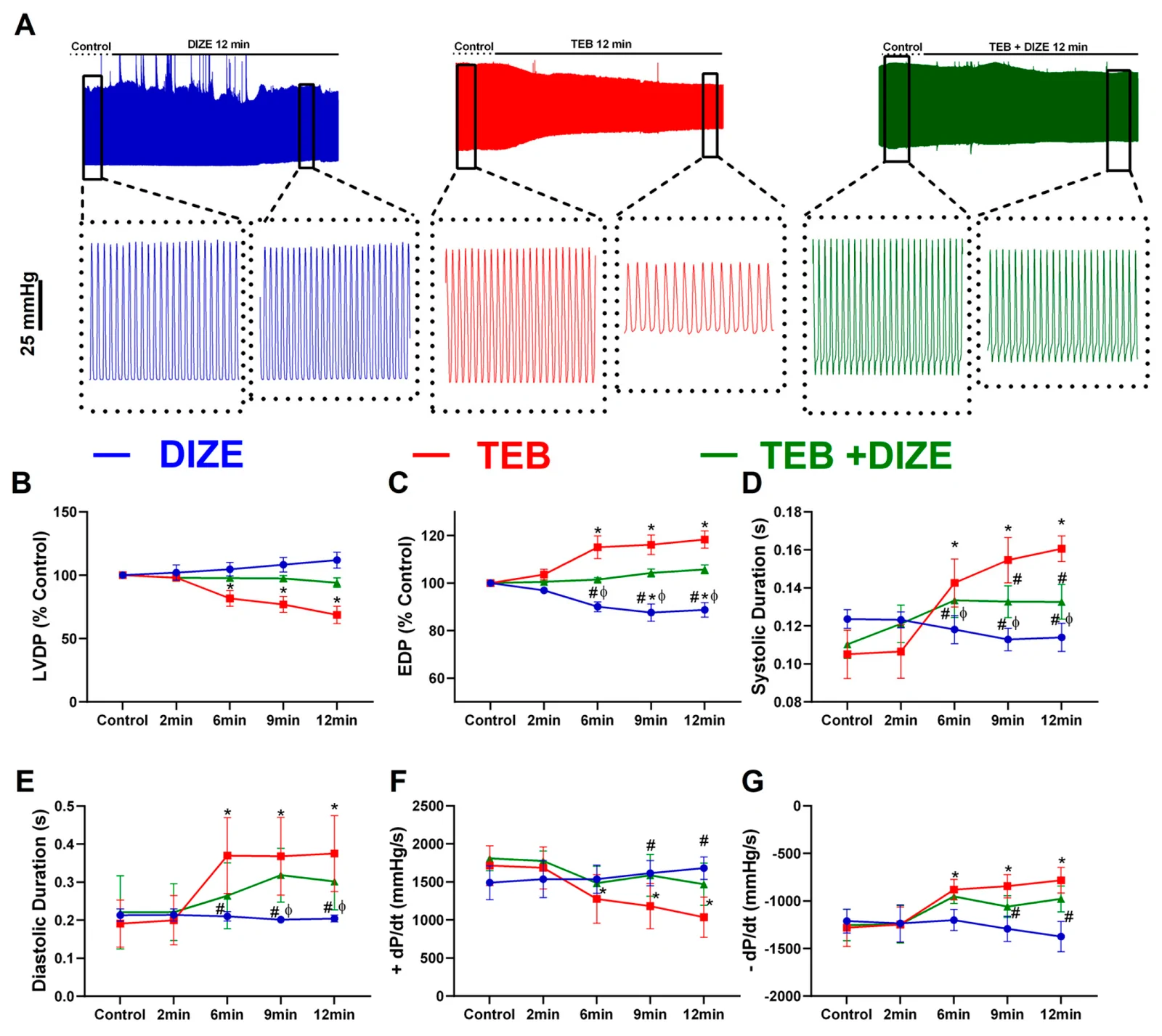
Representative recordings and quantitative analysis of left ventricular function in isolated hearts after Tebuconazole (TEB) and Diminazene (DIZE) treatment. Representative left ventricular pressure recordings (**A**). Time-course analysis of left ventricular developed pressure (LVDP) (**B**), end-diastolic pressure (EDP) (**C**), systolic duration (**D**), diastolic duration (**E**), maximal rate of pressure development (+dP/dt) (**F**), and maximal rate of pressure decay (−dP/dt) (**G**) during Langendorff perfusion with TEB or TEB + DIZE. Data are presented as mean ± SEM (*n* = 5 hearts per group). * *p* < 0.05 vs. baseline (2 min); # *p* < 0.05 vs. TEB; ɸ *p* < 0.05 vs. TEB + DIZE, two-way repeated-measures ANOVA followed by Bonferroni’s multiple comparisons test.

Representative traces from ventricular contractility are displayed in Figure 4A. Acute perfusion with TEB induced a progressive impairment of left ventricular mechanical function. LVDP gradually declined, becoming significantly reduced after 6 min of perfusion and reaching approximately 68% of baseline values at 12 min, corresponding to a 32% ± 6.73 reduction (Figure 4B). In parallel, TEB significantly increased end-diastolic pressure (EDP), indicating impaired ventricular relaxation and increased diastolic stiffness (Figure 4C). TEB also prolonged systolic duration from 6 min onward (Figure 4D) and modestly increased diastolic duration (Figure 4E). In addition, the maximal rate of pressure development (+dP/dt) progressively declined during TEB perfusion (Figure 4F), consistent with impaired myocardial contractility, whereas the maximal rate of relaxation (−dP/dt) was not significantly affected (Figure 4G). Concurrent treatment with DIZE markedly attenuated these alterations. Hearts treated with TEB + DIZE maintained LVDP close to baseline throughout the experimental period, exhibited a significantly smaller increase in EDP, and showed preservation of systolic duration and +dP/dt compared with hearts exposed to TEB alone. Overall, these findings demonstrate that acute TEB exposure impairs both systolic and diastolic ventricular performance, whereas DIZE effectively preserves cardiac mechanical function.

### 3.5. DIZE Protects Myocardial Electrical Function

**Figure 5:**
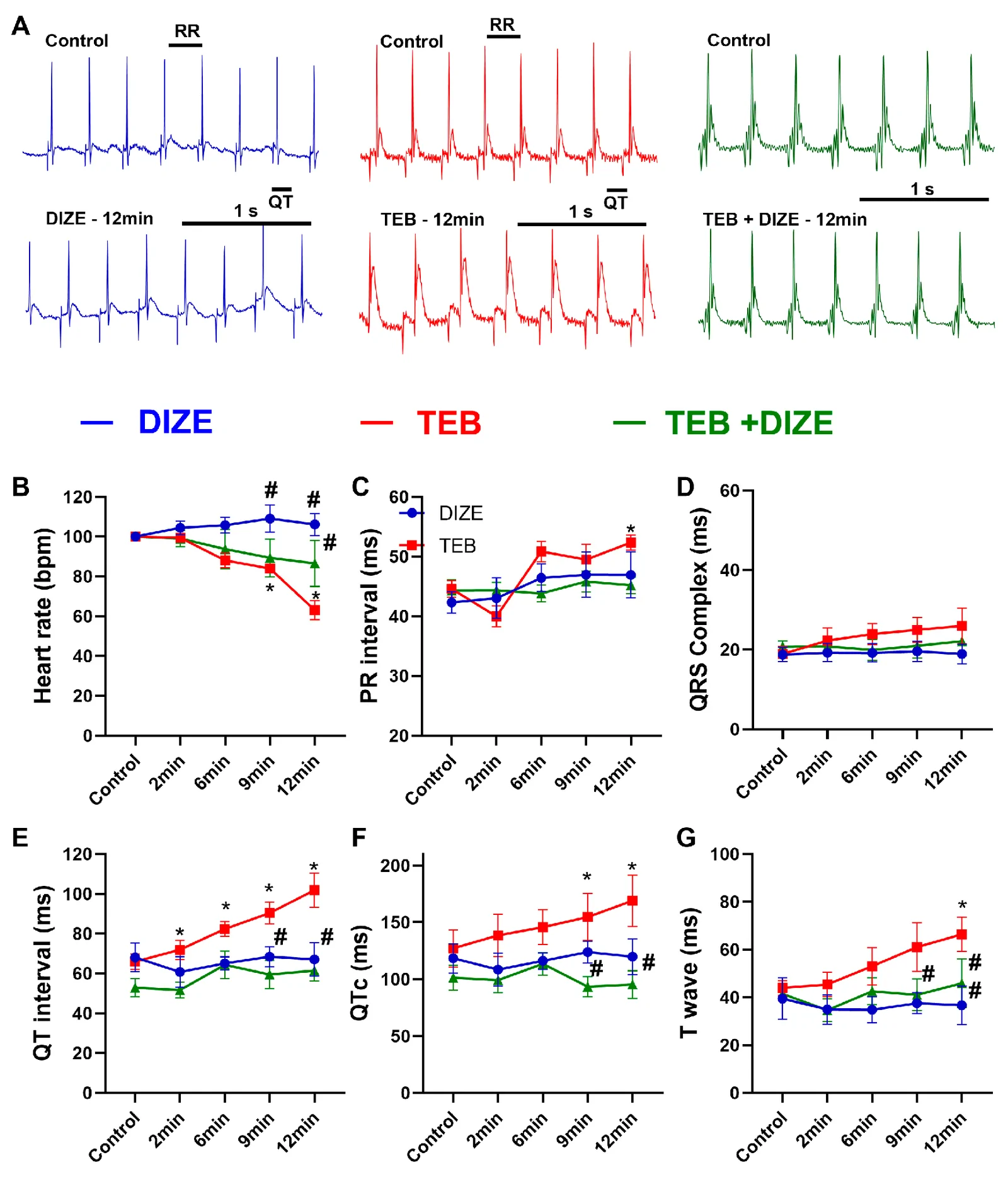
DIZE prevents TEB-induced alterations in cardiac electrical activity during Langendorff perfusion. Representative electrocardiographic recordings obtained at baseline (Control) and after 12 min of perfusion with DIZE, TEB, or TEB + DIZE (**A**). Time-course analysis of heart rate (**B**), PR interval (**C**), QRS duration (**D**), QT interval (**E**), corrected QT interval (QTc) (**F**), and T-wave duration (**G**). Acute TEB perfusion induced progressive bradycardia, prolonged PR, QT, and QTc intervals, and increased T-wave duration, whereas concurrent treatment with DIZE largely prevented these electrophysiological alterations. Data are presented as mean ± SEM (*n* = 5 hearts per group). * *p* < 0.05 vs. baseline (Control); # *p* < 0.05 vs. TEB; two-way repeated-measures ANOVA followed by Bonferroni’s multiple comparisons test.

Representative ECG traces are shown in Figure 5A. Acute perfusion with TEB induced marked alterations in cardiac electrical activity, as evidenced by the representative electrocardiographic recordings and quantitative analyses. Time-course analysis demonstrated a progressive reduction in heart rate, reaching approximately 38% after 12 min of perfusion relative to baseline (2 min) (Figure 5B). TEB also significantly prolonged the PR interval at 12 min compared with baseline (Figure 5C), indicating delayed atrioventricular conduction, whereas QRS duration exhibited only a slight, non-significant increase throughout the experimental period (Figure 5D). In addition, TEB significantly prolonged the QT and QTc intervals relative to baseline (Figure 5E,F), indicating delayed ventricular repolarization, and increased T-wave duration (Figure 5G), suggesting greater heterogeneity of the repolarization process. In contrast, concurrent treatment with DIZE largely prevented these electrophysiological alterations. In the TEB + DIZE group, heart rate remained stable throughout the protocol, and the PR, QT, QTc, and T-wave intervals showed no significant changes compared with baseline. Collectively, these findings demonstrate that acute TEB exposure induces bradycardia, delays atrioventricular conduction, and prolongs ventricular repolarization, whereas DIZE preserves myocardial electrical stability and prevents the electrophysiological disturbances induced by TEB.

## 4. Discussion

**Figure 6:**
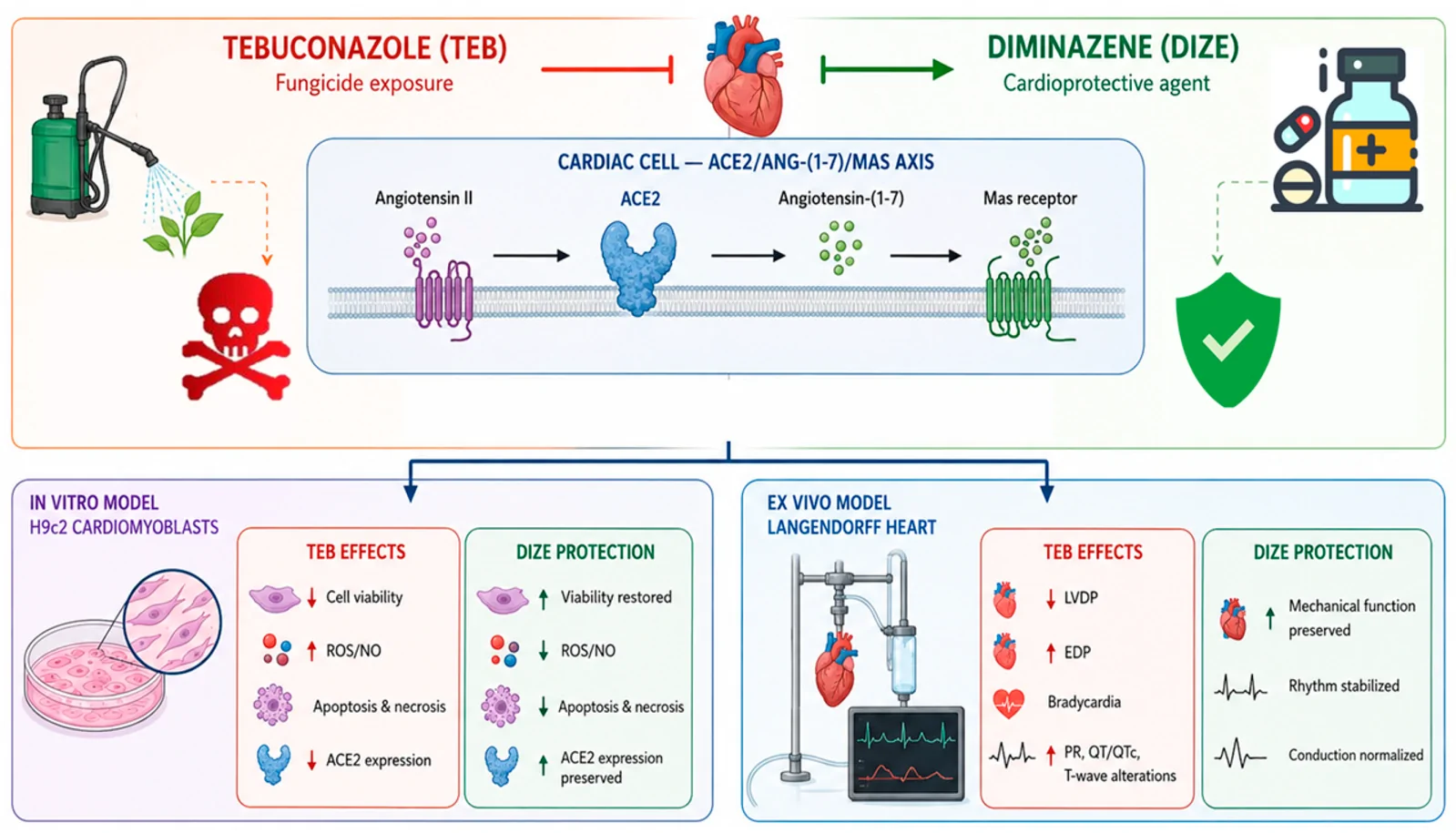
Tebuconazole (TEB)-induced cardiotoxicity and cardioprotection by diminazene (DIZE). In H9c2 cardiomyoblasts, TEB exposure was associated with reduced cell viability, increased oxidative/nitrosative stress (ROS/NO), apoptosis and necrosis, and showed a tendency toward reduced ACE2 expression, although DIZE does not directly demonstrate activation of the entire pathway. In the ex vivo Langendorff heart model, TEB impaired cardiac mechanical and electrical function, characterized by reduced left ventricular developed pressure (LVDP), increased end-diastolic pressure (EDP), bradycardia, and alterations in PR and QT/QTc intervals and T-wave morphology. DIZE preserved ACE2 expression, restored cellular viability, attenuated oxidative/nitrosative stress and cell death, and improved cardiac mechanical, rhythm, and conduction parameters. Abbreviations: ACE2, angiotensin-converting enzyme 2; Ang-(1–7), angiotensin-(1–7); DIZE, diminazene; EDP, end-diastolic pressure; LVDP, left ventricular developed pressure; Mas, Mas receptor; NO, nitric oxide; PR, PR interval; QTc, corrected QT interval; ROS, reactive oxygen species; TEB, tebuconazole. The upward arrow (↑) indicates an increase, whereas the downward arrow (↓) indicates a decrease in the corresponding parameter.

The present study provides convergent cellular and ex vivo evidence that TEB exerts a direct and multifaceted cardiotoxic effect, while DIZE confers cardioprotection in our experimental models, although our findings do not directly demonstrate activation of the entire pathway by DIZE, as shown in Figure 6. Our findings demonstrate that TEB induces dose-dependent cytotoxicity, characterized by increased apoptosis and necrosis, accompanied by marked oxidative and nitrosative stress, ventricular mechanical dysfunction, and profound electrical instability. DIZE attenuated TEB-induced cell death and oxidative imbalance, and further prevented heart dysfunction at the whole-organ level, both in terms of ventricular contractility and myocardial electrical integrity. This profile suggests that DIZE may represent a promising pharmacological modulator of pesticide-induced cardiotoxicity.

Exposure to TEB significantly increased NO and ROS availability, reflecting a pronounced redox imbalance capable of triggering cell death in H9c2 cardiomyoblasts. This TEB-induced cardiotoxicity is consistent with the activation of apoptotic pathways and mitochondrial dysfunction, processes tightly linked to excessive free radical generation and oxidative damage in cardiac cells [8]. The simultaneous elevation of NO and ROS observed in our fluorescence assays indicates that TEB induces both oxidative and nitrosative stress, thereby favoring cell death signaling through apoptotic and necrotic pathways, and reinforcing their role as measurable indicators of toxic cellular responses.

This interpretation is consistent with previous evidence demonstrating that DIZE exerts direct mitochondrial actions, preserving ATP production, reducing proton leak, and limiting ROS generation in ischemia/reperfusion models [25]. As mitochondria represent the principal source of free radicals under chemical stress conditions such as TEB exposure, mitochondrial stabilization emerges as a central mechanism underlying DIZE-mediated cytoprotection. By preserving mitochondrial bioenergetics, DIZE likely reduces ROS generation and secondarily limits excessive NO accumulation, thereby preventing peroxynitrite formation and irreversible oxidative damage to cardiomyocytes, further supporting the central role of mitochondrial and redox-related parameters as mechanistic biomarkers in this model. Our findings also demonstrated that while TEB alone tended to reduce ACE2 expression, the addition of DIZE modulated this effect, maintaining ACE2 at levels comparable to the control.

Considering the ex vivo analyses, the marked reduction in LVDP, elevation of EDP, prolongation of systolic duration, and depression of +dP/dt observed after acute TEB exposure indicate a profound impairment of ventricular mechanics, reflecting disruption of excitation–contraction coupling (ECC). We have previously demonstrated that TEB can block L-type Ca^2+^ currents [9], which is consistent with the reduction in LVDP and the reduced intrinsic heart rate observed in isolated heart preparations, leading to impaired ventricular mechanical performance. The concomitant increase in EDP further suggests impaired lusitropy. Given the described effect of TEB in impairing cellular energetics [8], we suggest and hypothesized that increased EDP might result from reduced SERCA2a activity, phospholamban dysregulation, and mitochondrial energetic failure, which compromise ATP-dependent Ca^2+^ reuptake and myocardial relaxation [32,33].

In addition, a congested heart following the reduced LVDP could further potentiate this effect of increased EDP. These alterations are mechanistically coherent with oxidative and nitrosative stress, since ROS and reactive nitrogen species directly modify RyR2, SERCA2a, and contractile proteins, leading to Ca^2+^ leak, reduced Ca^2+^ transient amplitude, and inefficient ECC [34,35]. The ability of DIZE to preserve LVDP, normalize EDP, prevent systolic prolongation, and restore +dP/dt might suggest protection of Ca^2+^ cycling dynamics and myocardial bioenergetics. This protective effect is consistent with the stabilization of mitochondrial function and ATP production, which are essential for SERCA2a activity and cross-bridge detachment kinetics [36,37,38].

Previous work from our group has established that TEB possesses a dangerous arrhythmogenic potential [12]. Specifically, we have demonstrated that TEB directly blocks repolarizing potassium currents, which delays cardiac repolarization, extends action potential duration (APD), and subsequently prolongs the QT interval [11,12]. In the present study, DIZE effectively counteracted these electrical disruptions, successfully normalizing both heart rate and the QTc interval. This aligns with our prior findings confirming the powerful cellular and ex vivo antiarrhythmic properties of DIZE [27]. Although the precise single-channel cellular bases were not directly recorded in this specific protocol, we speculate and hypothesized that the protective electrical phenotype driven by DIZE involves potassium currents intrinsically tied to the metabolic health of the cardiomyocyte. A major potential candidate is the ATP-sensitive potassium channel (KATP), which our group has previously shown to be upregulated or activated by DIZE therapy [23]. We speculate that by potentially sustaining currents like KATP alongside its antioxidant profile, DIZE prevents excessive APD prolongation and substrate heterogeneity under toxic stress.

The combination of impaired ventricular contractility, diastolic dysfunction, and prolonged ventricular repolarization constitutes a classic substrate for malignant arrhythmias and mechanical–electrical instability, particularly under conditions of sympathetic stimulation or metabolic stress [39]. Prolongation of QT/QTc intervals, bradycardia, and PR interval delay are well-established predictors of ventricular arrhythmias and sudden cardiac death, especially in structurally or metabolically compromised myocardium [40].

It is important to highlight that the widespread agricultural use and environmental persistence of TEB raise relevant public health concerns, as chronic low-dose exposure may contribute to cumulative cardiovascular risks in exposed populations, particularly agricultural workers and rural communities [41]. Within this context, the ability of DIZE to preserve myocardial mechanical performance and electrical stability in the present experimental models suggests a potential cardioprotective effect against TEB-induced cardiac injury. However, further studies using chronic exposure models, in vivo approaches, and clinically relevant exposure scenarios are required to determine whether these findings can be translated into preventive or therapeutic strategies for environmentally induced cardiac dysfunction. Thus, our results provide initial evidence supporting the investigation of DIZE as a potential pharmacological candidate and highlight the relevance of drug repurposing approaches at the interface between environmental toxicology and cardiovascular research.

A physiologically based toxicokinetic model demonstrates that typical human exposures to TEB [6], such as dietary ingestion at the Acceptable Daily Intake (ADI) limit or occupational dermal exposure, result in simulated peak systemic levels ranging from approximately 1.0 to 6.2 µmol. Consequently, the concentrations used in our experimental design were intentionally supraphysiological. This high-concentration approach was adopted because it is necessary to reliably unmask the fundamental cellular, mechanical, and electrophysiological mechanisms underlying TEB-induced cardiotoxicity and the corresponding cardioprotective pathways of DIZE. Furthermore, investigating these extreme concentrations holds significant clinical and toxicological relevance for understanding and managing cases of severe acute intoxication, particularly in scenarios of intentional self-poisoning (suicide attempts), where systemic and tissue concentrations vastly exceed standard environmental or occupational exposure thresholds.

This study has some limitations that should be acknowledged. First, the experimental models employed, H9c2 cardiomyoblasts and isolated perfused hearts, do not fully recapitulate the complexity of in vivo cardiovascular physiology, particularly the influence of neurohumoral regulation, immune responses, vascular interactions, and chronic exposure dynamics. Although H9c2 cardiomyoblasts are widely used to investigate cardiotoxicity, oxidative stress, and cell death mechanisms, they do not fully recapitulate the phenotype of mature, electrically active adult cardiomyocytes, particularly with respect to electrophysiological properties. In addition, only male Wistar rats were used in the ex vivo experiments. Although this approach was based on previous studies and aimed to reduce biological variability, sex may influence cardiac responses to toxic exposure and pharmacological interventions. Second, the use of acute exposure paradigms limits the extrapolation of these findings to long-term, low-dose environmental exposure scenarios. In addition, our evaluation was restricted to cardiovascular outcomes, although TEB exposure is known to affect multiple organ systems. Consequently, the potential protective effects of this intervention on other target organs, such as the liver, kidneys, or brain, remain unknown. A more comprehensive assessment of systemic toxicity would provide a broader understanding of the translational potential of DIZE in pesticide poisoning. Therefore, future studies should investigate whether the cardioprotective effects observed herein are accompanied by protection against extra-cardiac toxicities, thereby establishing the overall therapeutic value of ACE2 activation in the management of TEB-induced toxicity.

Despite these limitations, this study provides integrated experimental evidence that TEB exposure induces coordinated cellular, mechanical, and electrical cardiac alterations, and that DIZE attenuates these effects under the experimental conditions evaluated. By demonstrating that DIZE contributes to the preservation of redox balance, ventricular mechanical performance, and electrical stability in cellular and ex vivo models, our findings improve the understanding of the mechanisms involved in TEB-induced cardiac injury and identify DIZE as a potential pharmacological candidate for further investigation. However, these findings should be interpreted as preliminary evidence, and additional studies involving chronic exposure models, in vivo approaches, pharmacokinetic evaluations, and clinically relevant conditions are required to determine the translational applicability of DIZE in the context of pesticide-associated cardiotoxicity. Collectively, this work provides mechanistic insights into environmentally induced cardiac injury and highlights drug repurposing as a promising research strategy for identifying potential interventions at the interface between toxicology and cardiovascular medicine.

## Data Availability

The data that support the findings of this study are available from the corresponding author upon reasonable request.
